# Mycosins Are Required for the Stabilization of the ESX-1 and ESX-5 Type VII Secretion Membrane Complexes

**DOI:** 10.1128/mBio.01471-16

**Published:** 2016-10-18

**Authors:** Vincent J. C. van Winden, Roy Ummels, Sander R. Piersma, Connie R. Jiménez, Konstantin V. Korotkov, Wilbert Bitter, Edith N. G. Houben

**Affiliations:** a Department of Medical Microbiology and Infection Control, VU University Medical Center, Amsterdam, The Netherlands; b Department of Medical Oncology, OncoProteomics Laboratory, VU University Medical Center, Amsterdam, The Netherlands; c Department of Molecular and Cellular Biochemistry, and Center for Structural Biology, University of Kentucky, Lexington, Kentucky, USA; d Section Molecular Microbiology, Amsterdam Institute of Molecules, Medicines and Systems, Vrije Universiteit Amsterdam, Amsterdam, The Netherlands

## Abstract

Pathogenic mycobacteria contain up to five type VII secretion (T7S) systems, ESX-1 to ESX-5. One of the conserved T7S components is the serine protease mycosin (MycP). Strikingly, whereas MycP is essential for secretion, the protease activity of MycP_1_ in *Mycobacterium tuberculosis* has been shown to be dispensable for secretion. The essential role of MycP therefore remains unclear. Here we show that MycP_1_ and MycP_5_ of *M. marinum* have similar phenotypes, confirming that MycP has a second unknown function that is essential for its T7S system. To investigate whether this role is related to proper functioning of the T7S membrane complex, we first analyzed the composition of the ESX-1 membrane complex and showed that this complex consists of EccBCDE_1_, similarly to what was previously shown for ESX-5. Surprisingly, while mycosins are not an integral part of these purified core complexes, we noticed that the stability of both the ESX-1 complex and the ESX-5 complex is compromised in the absence of their MycP subunit. Additional interaction studies showed that, although mycosins are not part of the central ESX membrane complex, they loosely associate with this complex. We hypothesize that this MycP association with the core membrane complex is crucial for the integrity and functioning of the T7S machinery.

## IMPORTANCE

Among the major virulence factors of pathogenic mycobacteria are the type VII secretion (T7S) systems. Three of these systems, ESX-1, ESX-3, and ESX-5, have been shown to be crucial for virulence or viability. Here we describe the function of mycosin proteases, which are conserved components within these systems. We show that MycP_1_ and MycP_5_ have a second, proteolytic-independent function which is essential for the T7S system. We additionally found that this second essential role is related to the stabilization and proper functioning of their respective ESX membrane core complexes. Finally, we found that this is mediated by a loose association of MycP with the complex. Understanding the essential role of mycosins in type VII secretion systems, which play central roles in the virulence and viability of pathogenic mycobacteria, may provide new intervention strategies to treat tuberculosis.

## INTRODUCTION

Pathogenic mycobacteria such as *Mycobacterium tuberculosis* and *Mycobacterium leprae* remain notorious human pathogens. Important virulence factors of pathogenic mycobacteria are the type VII secretion (T7S) systems and their substrates, which are required for the completion of the macrophage infection cycle and the uptake of nutrients and metabolites across its exceptionally hydrophobic and impermeable cell envelope (CE) (1–4). Pathogenic mycobacteria have up to five of these systems, called ESX-1 to ESX-5, of which ESX-1, ESX-3, and ESX-5 have been shown to be essential for virulence or bacterial viability (1, 5, 6).

ESX-1 is of pivotal importance for the virulence of pathogenic mycobacteria, with ESX-1 substrates being linked to phagosomal escape by destabilizing the phagosomal membrane of macrophages (1, 7). The importance of the ESX-1 system for virulence is also shown by the absence of part of the *esx-1* genomic locus in the vaccine strain *Mycobacterium bovis* BCG (8–10). This deletion is the major determinant for the attenuation of this strain. Also, in the fish pathogen *Mycobacterium marinum*, a close relative of *M. tuberculosis*, ESX-1 has been shown to mediate phagosomal escape and deletion of the *esx-1* region leads to a strong attenuation in zebrafish (11, 12).

The most recently evolved mycobacterial T7S system, ESX-5, is present only in the cluster of slow-growing mycobacteria. Interestingly, this cluster contains most of the pathogenic species. ESX-5 is responsible for the secretion of many proteins of the so-called proline-glutamic acid (PE) and proline-proline-glutamic acid (PPE) families and is linked to host immune modulation. In addition, ESX-5 has been shown to be essential for *in vitro* growth of *M. marinum* and *M. bovis* BCG by permeating the outer membrane to allow nutrient uptake (4, 13–15).

The ESX systems of mycobacteria share a set of conserved components (16, 17), five of which have one or more predicted transmembrane domains and are cell envelope localized (2). Four of these membrane proteins of the ESX-5 system, i.e., EccB_5_ to EccE_5_, form a large membrane complex of 1.5 MDa (2, 17, 18). Although crystal structures of the soluble domains of the individual components EccB, EccC, and EccD have been published previously (19, 20), there are currently no structural data for this complete membrane complex. Furthermore, the biochemical composition of this complex has been elucidated only for the ESX-5 system, whereas the composition and size of the other ESX complexes remain unknown.

The fifth conserved component with a predicted transmembrane domain is the subtilisin-like protease mycosin (MycP), which is among the most conserved T7S components (21). Although previous pulldown experiments indicated that MycP is not part of the core ESX membrane complex, MycP_3_ and MycP_5_ have been shown to be essential for mycobacterial viability and MycP_1_ and MycP_5_ are essential for ESX-1- and ESX-5-associated secretion, respectively (4, 22, 23). This indicates that each MycP is essential for and functions specifically within its respective ESX system. The crystal structures of the protease domains of MycP_1_ and MycP_3_ show a highly conserved overall subtilisin-like structure, with differences in the substrate binding groove indicating different substrate specificities (24, 25).

Surprisingly, thus far, only one substrate, ESX-1 substrate EspB, is known for any of the mycosins. This protein is processed by MycP_1_ *in vitro* and upon secretion by *M. tuberculosis* (22). Importantly, proteolytic activity of MycP_1_ is, however, not essential for ESX-1-associated secretion; a catalytically inactive MycP_1_ mutant of *M. tuberculosis* even showed increased secretion of ESX-1 substrates (22). Therefore, the essential function of mycosins in secretion remains unknown. The catalytically inactive MycP_1_ mutant of *M. tuberculosis* additionally showed decreased virulence in mice, but it is still unknown whether this is a direct effect of the deficiency in EspB processing or due to the observed increased secretion of ESX-1 substrates in this mutant. Together, these observations suggest a dual role of MycP within T7S, with MycP_1_ being essential for ESX-1 secretion whereas proteolytic activity of MycP_1_ is not essential for this function.

To further elucidate the dual function of mycosin proteases, we investigated MycP_1_ and MycP_5_ functioning in *M. marinum*. We show that, similarly to the ESX-1 system in *M. tuberculosis*, ESX-1- and ESX-5-mediated secretion is independent of the (predicted) proteolytic activity of their respective mycosins. However, we show that both the ESX-1 and ESX-5 membrane complexes are not stable in the absence of MycP_1_ and MycP_5_, respectively, providing an explanation of why mycosins are essential components in the T7S system.

## RESULTS

### MycP_1_ is essential for ESX-1-dependent secretion in *M. marinum*, while its protease activity is not.

**FIG 1:**
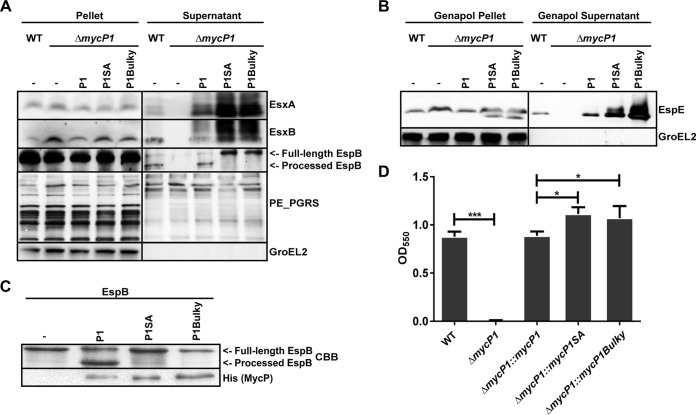
MycP_1_ is essential for ESX-1-dependent secretion in *M. marinum*, while proteolytic inactive MycP_1_ shows increased ESX-1 activity. (A) Immunoblot analysis of cell pellets and supernatants of wild-type (WT) *M. marinum* and the *mycP_1_* deletion strain complemented with a WT *mycP_1_* (P1) gene, a *mycP_1_* gene containing an active site mutation (P1SA), and a *mycP_1_* gene with a bulky residue in the substrate pocket (P1Bulky). Proteins were visualized with anti-EsxA, anti-CFP-10, and anti-EspB (ESX-1 substrates). As a control, blots were incubated with antibodies directed against the ESX-5 secreted PE_PGRS proteins and the cytosolic GroEL2 protein. (B) Immunoblot detection of cellular (Genapol Pellet) and surface-localized (Genapol Supernatant) proteins of the *M. marinum* WT strain and various *mycP_1_* mutants. Surface-localized proteins were extracted with Genapol X-080 and stained for the ESX-1 substrate EspE. (C) SDS-PAGE of an *in vitro* cleavage assay of EspB_mtub_ by WT MycP_1mth_ (P1), the active site mutant (P1SA), and the bulky mutant (P1Bulky). EspB was visualized by Coomassie brilliant blue (CBB) staining, and MycP1 was analyzed with immunoblotting and stained with anti-His. (D) Hemolysis detection of erythrocytes by the *M. marinum* WT strain and various *mycP_1_* mutants. Hemolysis was quantified by determining the OD_550_ absorption of the released hemoglobin. Statistical significant differences between strains were determined with one-way ANOVA; *n* = 6 per strain. *, *P* ≤ 0.05; ***, *P* ≤ 0.001.

To confirm the dual role of MycP_1_ in another species (22), we first deleted the *mycP_1_* gene of *M. marinum* via allelic exchange and confirmed that the gene was successfully deleted via PCR analysis (unpublished observation). As expected, this knockout mutant was no longer able to secrete the ESX-1 substrates EsxA, EsxB, and EspB. All examined substrates were still detected in the pellet fractions (Fig. 1A). The ESX-1 substrate EspE was also no longer extractable from intact cells by the mild detergent Genapol X-080 (Fig. 1B), indicating that it was no longer present on the cell surface. ESX-1-dependent secretion was fully complemented by the introduction of the wild-type gene (P1; Fig. 1A and B). To assess the role of the protease activity of MycP_1_ in secretion, we complemented the Δ*mycP_1_* mutant also with a proteolytically inactive version, *mycP_1_*::*S354A* (P1SA). In agreement with a previous study (22), expression of proteolytic inactive MycP_1_ resulted in increased secretion of EsxA and EsxB (Fig. 1A). We also observed an increase in the amount of surface-localized EspE (Fig. 1B). Whereas the wild-type strain showed efficient processing of EspB, mainly full-length EspB was detected in the supernatant of the S354A mutant (Fig. 1A), which is consistent with the effect observed for the MycP_1_ active site mutant of *M. tuberculosis* (22). There was no increase in the ESX-5-dependent secretion of proteins of the PE subfamily with polymorphic GC-rich repetitive sequences (PE_PGRS), showing that the proteins are not in general more efficiently secreted by the MycP_1_ proteolytically inactive mutant.

The secretion of ESX-1 substrates has been shown to be essential for contact-dependent lysis of erythrocytes by *M. marinum*, which serves as a model for the ESX-1-dependent lysis of phagosomal membranes and thus for mycobacterial virulence (11). We confirmed that our wild-type *M. marinum* strain was capable of lysing erythrocytes, whereas the Δ*mycP_1_* mutant showed no hemolytic activity (Fig. 1D). We could restore this lysing capability by complementing the mutant with both wild-type *mycP_1_* and the proteolytically inactive MycP_1_ mutant. The latter complementation resulted in significantly increased hemolysis activity compared to that seen with wild-type cells, which is in line with the increased secretion of ESX-1 substrates in this mutant. Together, these data show that, in the presence of a proteolytically inactive MycP_1_ variant, the ESX-1 system is more active. Finally, we created a version of MycP_1_ where the access to the active site is partially blocked by placing a bulky amino acid, i.e., a tyrosine, at different positions in the substrate binding groove. We first analyzed the effect of these mutations on the ability of MycP_1_ of *Mycobacterium thermoresistibile* (MycP_1mth_) to cleave its substrate EspB *in vitro* (25). Introducing a tyrosine at position 239, generating *mycP_1mth_*::*N239Y*, completely blocked protease activity (Fig. 1C). Next, we investigated the effect of the *mycP_1mth_*::N239Y mutation (N259Y in *M. marinum*) on secretion and hemolysis by *M. marinum*. The bulky mutant (P1Bulky) showed a phenotype similar to that of the active site mutant (Fig. 1A, B, and D), with the bacteria still capable of oversecreting ESX-1 substrates and a more efficient lysis of erythrocytes. This indicates that in addition to protease activity, substrate binding to MycP_1_ is also not essential for secretion.

### MycP_5_ shows a phenotype similar to that of MycP_1_.

**FIG 2:**
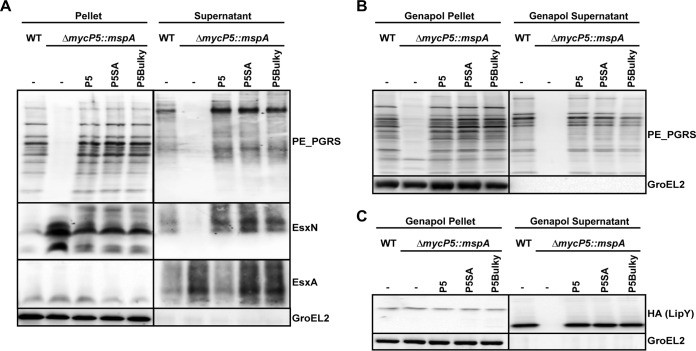
MycP_5_ is required for secretion but can be complemented with variants that have mutations in the active site or at the binding site. (A) Immunoblot analysis of cell pellets and supernatants of wild-type (WT) *M. marinum* and the *mycP_5_* deletion strain complemented with a WT *mycP_5_* (P5) gene, a *mycP_5_* gene containing an active site mutation (P5SA), and a *mycP_5_* gene with a bulky residue in the substrate pocket (P5Bulky). Blots were stained for ESX-5 substrates with anti-PE_PGRS and anti-EsxN. As a loading control, blots were probed with antibodies directed against the ESX-1 substrate EsxA and cytosolic GroEL2. (B) Detection of cellular (Genapol Pellet) and cell surface-localized (Genapol Supernatant) PE_PGRS proteins of the *M. marinum* wild-type (WT) strain and the *mycP_5_* deletion strain complemented with various *mycP_5_* mutant genes by immunoblotting. (C) Immunoblot detection of cellular (Genapol Pellet) and cell surface-localized (Genapol Supernatant) proteins of wild-type (WT) *M. marinum* and the various *mycP_5_* mutant strains expressing C-terminal HA-tagged LipY.

Ates et al. (4) previously showed that a *mycP_5_* transposon mutant in *M. marinum* is no longer able to secrete the ESX-5 substrate group of PE_PGRS proteins. In this study, we confirmed this secretion defect for an *M. marinum mycP_5_* knockout strain (Fig. 2) and that the original phenotype could be restored by complementation with wild-type *mycP_5_* (P5; Fig. 2). Similarly to the phenotype of MycP_1_, complementation of the *mycP_5_* knockout strain with the predicted proteolytically inactive mutant *mycP_5_*::*S461A* (P5SA) or the bulky mutant *mycP_5_*::*D362Y* (P5Bulky) fully restored ESX-5-dependent secretion. However, we did not observe an increase in the secretion of ESX-5 substrates, such as the PE_PGRS proteins or EsxN and LipY. Importantly, LipY, which is normally processed upon secretion (26), was processed in the active site and bulky mutants in a manner similar as seen in the wild-type strain. Also, the pattern seen with the PE_PGRS proteins, which are potentially processed upon secretion, was unaltered. Either MycP_5_ is not involved in the processing of these substrates or there is redundancy in the protease activities. In conclusion, MycP_5_ is also essential for protein secretion via ESX-5, but this function is not linked to its putative protease activity.

### The composition of the ESX-1 membrane complex is similar to that of the ESX-5 membrane complex.

Because MycP is probably an inner membrane protein, we hypothesized that MycP may be involved in the correct functioning of the core membrane complex of T7S systems. We have shown previously by blue native PAGE (BN-PAGE) and antibody (Ab) pulldown experiments that the membrane complex of the ESX-5 system has a size of 1.5 MDa and consists of four conserved membrane proteins, i.e., EccB_5_, EccC_5_, EccD_5_, and EccE_5_ (2); no MycP_5_ could be detected in these purified samples. Here, we set out to improve the purification procedure by the introduction of an affinity tag, not only to more accurately detect less-abundant components of the ESX-5 membrane complex but also to determine the composition of the ESX-1 membrane complex.

**FIG 3:**
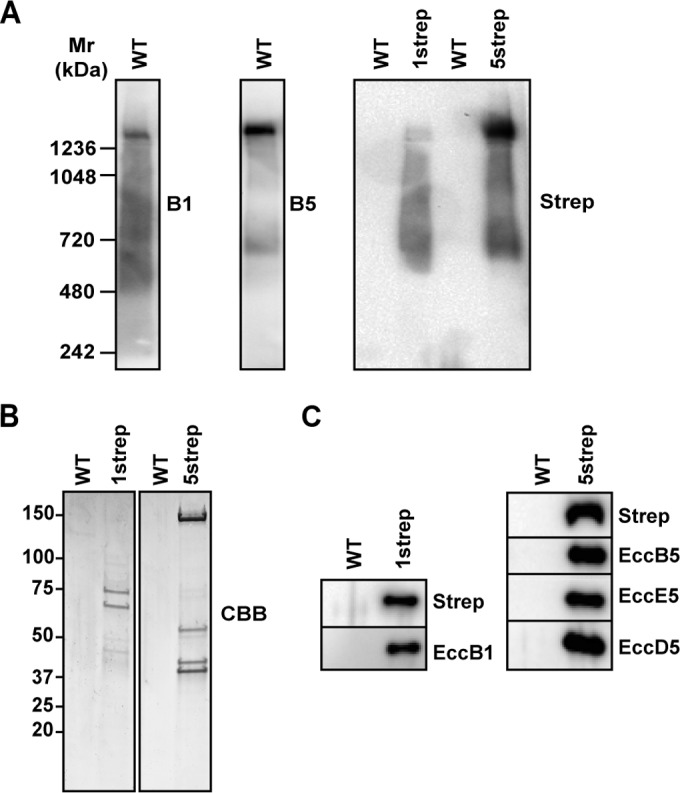
Isolation and characterization of the ESX-1 membrane complex, whose composition is similar to that of the ESX-5 membrane complex. (A) Immunoblot of detergent-solubilized cell envelope fractions of the *M. marinum* wild-type (WT), *eccCb_1_*::*tn-eccCb_1_-2strep* (1strep), and Δ*eccC_5_-eccC_5_-2strep* (5strep) strains after blue native polyacrylamide gel electrophoresis. Blots were stained with anti-EccB_1_ (B_1_), anti-EccB_5_ (B_5_), or anti-Strep tag (strep) antibodies as indicated. (B) SDS-PAGE analysis and Coomassie staining of the Strep-Tactin-purified ESX-1 membrane complex from *M. marinum eccCb_1_*::*tn-eccCb_1_-2strep* and ESX-5 membrane complex from *M. marinum* Δ*eccC_5_-eccC_5_-2strep*. Purifications using WT *M. marinum* strains served as a negative control. Isolated proteins were analyzed by mass spectrometry. (C) Immunoblot analysis of the purified ESX-1 membrane complex from *M. marinum eccCb_1_*::*tn-eccCb_1_-2strep* and ESX-5 membrane complex from *M. marinum* Δ*eccC_5_-eccC_5_-2strep*.

As the ESX-1 complex had not been analyzed before, we first investigated whether the ESX-1 system of *M. marinum* forms a similar complex. To analyze this, we generated polyclonal antibodies directed against the C-terminal fragment of EccB_1_. These antibodies were used to identify the ESX-1 membrane complex in *n*-dodecyl β-d-maltoside (DDM)-solubilized membrane proteins separated on BN-PAGE, as was done previously for ESX-5. EccB_1_ antibodies stained a number of different complexes, the largest of which was approximately 1.5 MDa, similarly to ESX-5 (Fig. 3A). To be able to isolate the complex, we introduced EccCb_1_ containing a C-terminal Twin-Strep-tag in an *eccCb_1_* transposon mutant of *M. marinum* (27). The affinity tag did not interfere with EccCb_1_ functioning, as introduction of the construct fully restored ESX-1-dependent secretion (see Fig. S1 in the supplemental material). The tag also did not interfere with formation of the 1.5-MDa ESX-1 membrane complex as shown by BN-PAGE analysis of detergent-solubilized membrane fractions and immunoblotting using Strep antibodies (Fig. 3A), although these complexes were less pronounced (Fig. 3A).

We subsequently performed pulldown experiments on detergent-solubilized membrane fractions using Strep-Tactin beads. We observed a number of copurified proteins after Coomassie staining (Fig. 3B), and we confirmed that two were EccCb_1_-Strep and EccB_1_ by immunoblot analysis (Fig. 3C). To identify the other copurified proteins, liquid chromatography-tandem mass spectrometry (LC-MS/MS) analysis was performed on the complete Strep-tag-purified sample. This analysis showed the significantly increased presence of EccB_1_, EccCa_1_, EccCb_1_, EccD_1_, EccE_1_, and a hypothetical protein, MMAR_2712 (Table 1), in the samples containing EccCb_1_-Strep but not in control samples containing purified material from solubilized membranes of wild-type *M. marinum*. The presence of MMAR_2712 was surprising and might indicate the presence of an additional component. However, homology and structure predictions (Phyre^2^) indicated that MMAR_2712 is a transmembrane protein with a large periplasmic domain containing a predicted phosphate binding site, an activity unrelated to ESX-1 functioning. Furthermore, the gene encoding this protein is highly conserved in many bacterial species without an ESX-1 system. To test a possible interaction of this protein with the ESX complex, we introduced an N- or a C-terminal hemagglutinin (HA) tag in MMAR_2712 and isolated this protein using HA antibody beads. Subsequently, we used immunoblotting to determine if ESX-1 components were copurified. However, this experiment failed to confirm any interaction of MMAR_2712 with ESX-1 components (unpublished observations). From these combined observations, we conclude that it is unlikely that MMAR_2712 is a component of ESX-1. As with the previously analyzed ESX-5 membrane complex, we were not able to detect significantly more MycP_1_ peptides in the purified ESX-1 membrane complex, although a few specific MycP_1_ spectral counts were observed in this analysis. We therefore conclude that the composition and the size of the ESX membrane complex are conserved between the systems.

**TABLE 1:** Proteins copurified with EccCb_1_strep^a^

Identified protein	Protein description	MW	Sequence coverage (%)	MS/MS normalized spectral count				Fold change	*P* value	NSAF	
				WT		*eccCb_1_-strep*					
				A	B	A	B			A	B
EccCa_1_	ESX-1 core component	80.8	73.5	20	3	271	157	19.0	2.5 × 10^−3^	0.35	0.3
EccCb_1_	ESX-1 core component	64.6	49.2	7	0	172	98	37.5	2.4 × 10^−3^	0.40	0.38
EccB_1_	ESX-1 core component	51.3	75.5	7	3	85	53	13.9	1.4 × 10^−3^	0.37	0.38
EccE_1_	ESX-1 core component	50.9	64.7	11	1	76	46	10.1	4.6 × 10^−3^	0.20	0.15
MMAR_2712	Hypothetical protein	76.1	51.4	9	1	70	44	11.0	3.0 × 10^−3^		
EccD_1_	ESX-1 core component	51.3	14	0	0	18	11	∞	9.7 × 10^−4^	0.05	0.03
MycP1	ESX-1 component	47.7	41.7	0	0	11	8	∞	1.2 × 10^−3^		

a LC-MS/MS was performed on Strep-tag-purified material from *M. marinum* wild-type (negative control) and *M. marinum-eccCb_1_*::*tn-eccCb_1_strep* cell envelope fractions, followed by a two-way analysis. Proteins that showed >10 normalized spectral counts in both *eccCb_1_strep* pulldown samples and a normalized spectral abundance factor (NSAF) of >0.02 were selected. Data in columns A and B represent results from biological replicates. MW, molecular weight.

Next, we also modified the ESX-5 system with a Twin-Strep-tag to allow more-efficient purification of the ESX-5 membrane complex. For this, we complemented the previously characterized *M. marinum eccC_5_* knockout strain (4) with EccC_5_ containing a C-terminal Twin-Strep-tag. This affinity tag did not interfere with ESX-5-dependent secretion (see Fig. S1 in the supplemental material) or with formation of the 1.5-MDa ESX-5 membrane complex (Fig. 3). The Strep-tag purification of EccC_5_-Strep was significantly more efficient than the EccCb_1_-Strep purification and resulted in the copurification of the three known interactors, i.e., EccB_5_, EccD_5_, and EccE_5_, as shown by immunoblot analysis and LC-MS/MS analysis (Table 2). The mass spectrometry analysis revealed that, in this preparation also, there were no additional proteins copurified with EccC_5_; although spectral counts for MycP_5_ could be detected, these numbers were not above the spectral count threshold levels (Table 2). We therefore conclude that the mycosins are probably not a stable integral part of the ESX membrane complex.

**TABLE 2:** Proteins copurified with EccC_5_strep^a^

Identified protein	Protein description	MW	Sequence coverage (%)	MS/MS normalized spectral count				Fold change	*P* value	NSAF	
				WT		*eccC_5_-strep*					
				A	B	A	B			A	B
EccC_5_	ESX-5 core component	152.6	71.4	75	50	706	651	10.8	5.7 × 10^−6^	0.42	0.41
EccD_5_	ESX-5 core component	53.5	29.2	24	15	118	111	5.9	4.4 × 10^−5^	0.20	0.20
EccE_5_	ESX-5 core component	44.0	57.6	17	8	103	110	8.5	4.3 × 10^−5^	0.21	0.24
EccB_5_	ESX-5 core component	54.1	55.4	22	16	95	84	4.6	8.3 × 10^−5^	0.16	0.15
MycP_5_	ESX-5 component	59.8	29.5	0	3	9	19	6.9	4.8 × 10^−3^		

a LC-MS/MS was performed on Strep-tag-purified material from *M. marinum* wild-type (negative control) and *M. marinum-ΔeccC_5_-eccC_5_strep* cell envelope fractions, followed by a two way analysis. Proteins that showed >10 normalized spectral counts in both *eccC_5_strep* pulldown samples and a normalized spectral abundance factor (NSAF) of >0.05 were selected. The NSAF was calculated by dividing the normalized spectral counts from the nanoLC-MS/MS experiment by the relative molecular weight (*M*_r_) to obtain the spectral abundance factor (SAF) for each protein. Subsequently, each SAF was normalized by dividing it by the sum of the SAFs of the proteins in the complex. Data in columns A and B represent results from biological replicates. MW, molecular weight.

### MycP_1_ and MycP_5_ are involved in the stability of the ESX membrane complexes.

**FIG 4:**
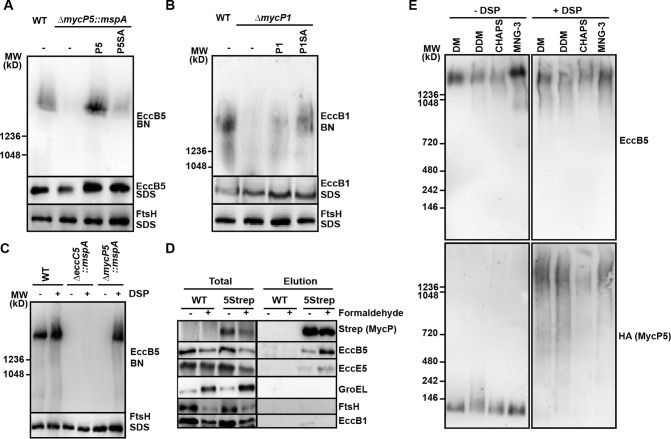
MycP_1_ and MycP_5_ are essential for ESX membrane complex stability. (A) Immunoblot analysis of detergent-solubilized cell envelope fractions of wild-type (WT) *M. marinum* and the *mycP_5_* deletion strain complemented with various *mycP_5_* mutant genes after BN-PAGE (BN) or SDS-PAGE (SDS). Blots were probed with antibodies directed against EccB_5_ and FtsH. (B) Immunoblot analysis of detergent-solubilized cell envelope fractions of wild-type (WT) *M. marinum* and the *mycP_1_* deletion strain complemented with various *mycP_1_* mutant genes after BN-PAGE (BN) or SDS-PAGE (SDS). Blots were stained with antibodies directed against EccB_1_ and FtsH. (C) Immunoblot analysis of DSP-cross-linked (+DSP) or DMSO-only-treated (−DSP) detergent-solubilized cell envelope fractions of the *M. marinum* wild-type (WT) strain, an *eccC_5_* deletion strain, and a *mycP_5_* deletion strain after BN-PAGE (BN) or SDS-PAGE (SDS). (D) Immunoblot analysis of solubilized cell envelope fractions (Total) and of proteins copurified with Strep-Tactin-purified MycP_5_-Strep (P5-Strep). Pulldown experiments using WT *M. marinum* material served as a negative control. (E) Immunoblot analysis of DSP-cross-linked (+DSP) or DMSO-only-treated (−DSP) detergent-solubilized cell envelope fractions of the *M. marinum mycP_5_* deletion strain complemented with HA-tagged MycP_5_ after BN-PAGE (BN). DM, *n*-decyl-β-d-maltopyranoside; DDM, *n*-dodecyl β-d-maltoside; CHAPS, 3-[(3-cholamidopropyl)-dimethylammonio]-1-propanesulfonate; MNG-3, maltose neopentyl glycol-3.

Although mycosins do not appear to be part of the core complex, the mycosins might still be involved in the correct functioning of this membrane complex. To analyze this, we first analyzed the presence of the ESX-5 membrane complex in the absence or presence of MycP_5_. While the 1.5-MDa membrane complex was readily visualized on BN-PAGE using polyclonal antibodies directed against EccB_5_, EccC_5_, and EccD_5_ for the wild-type strain, the complex levels were strongly reduced when membrane proteins of the *mycP_5_* knockout mutant were analyzed (Fig. 4A; see also Fig. S2A in the supplemental material). Complementation with both wild-type *mycP_5_* and *mycP_5_*::*S461A* restored the complex to wild-type levels. This effect on complex formation was not due to decreased stability of the separate subunits, as the expression levels of EccC_5_ and EccD_5_ were not affected, whereas EccB_5_ levels were reduced only slightly upon the *mycP_5_* deletion. A similar effect was observed for the ESX-1 complex; we could detect the 1.5-MDa ESX-1 complex in the wild-type strain, while this complex was not observed in the absence of MycP_1_ (Fig. 4B). This phenotype could be complemented by introduction of wild-type MycP_1_ or the active site mutant. Expression levels of EccB_1_ were not affected by knocking out *mycP_1_*. These results suggest that the strongly reduced membrane complex levels in the absence of the respective mycosins were not due to diminished expression of individual membrane components.

The observation that small amounts of the 1.5-MDa ESX-5 membrane complex could be detected in the *mycP_5_* knockout strain suggested that the membrane complex is less efficiently formed or is less stable. To distinguish between these two possibilities, we treated half of the membrane fractions with the cross-linking agent dithiobis(succinimidyl propionate) (DSP) before solubilization was performed to fix protein-protein interactions. DSP cross-linking did not affect ESX-5 complex levels in the wild-type strain. Also, in the negative-control strain, i.e., the *eccC_5_* knockout mutant, the presence of DSP did not restore complex formation of the remaining components. In contrast, cross-linking had a major effect in the *mycP_5_* mutant, as the ESX-5 membrane complex was detectable at wild-type levels in the DSP-treated membranes (Fig. 4C). A similar experiment was performed for ESX-1. Also, in this case DSP treatment of *mycP_1_* knockout membranes resulted in a stabilizing effect on the ESX-1 complex (see Fig. S2B in the supplemental material). This shows that the conserved components of the ESX-1 and ESX-5 membrane complex interact in principle and seem to properly form the ~1.5-MDa membrane complex in the absence of their MycP component but that the complexes more easily dissociate after detergent extraction. We therefore conclude that the mycosins of ESX-1 and ESX-5 are involved in stabilization of the respective membrane complexes. We propose that this stabilization is crucial for membrane complex functioning, explaining the essential role of MycP in the T7S system.

### MycP_5_ is associated with the ESX-5 core complex.

In the mass spectrometry results from the purified EccCb_1_ and EccC_5_ protein samples, we did observe a few specific spectral counts for MycP_1_ and MycP_5_, respectively. These counts were too low to conclude that MycP is a stable component of the T7S membrane complex. However, we hypothesized that MycP could be loosely associated with the complex and could thereby stabilize the core complex. To investigate this, we tried to preserve this interaction by testing different mild detergents to solubilize the cell envelope proteins of the *mycP_5_* deletion strain complemented with MycP_5_ containing a C-terminal HA tag for detection. The HA tag did not interfere with ESX-5-dependent secretion and therefore did not affect MyP_5_ functioning (see Fig. S1C in the supplemental material). Although several detergents did show solubilization comparable to that seen with DDM, they did not preserve the interaction of the ESX-5 complex with MycP_5_ (Fig. 4E). Next, we tried to fix the interaction by treating the cell envelope fractions with DSP prior to the detergent extraction. Interestingly, the DSP treatment resulted in a shift of the HA-tagged MycP_5_ to a molecular weight corresponding to the ESX-5 core complex, indicating that MycP_5_ is associated with the ESX-5 complex after cross-linking. To confirm that cross-linking stabilizes the interaction, we performed Strep-Tactin pulldown experiments on cross-linked and detergent-solubilized membrane fractions of either the wild-type strain or a *mycP_5_* deletion strain complemented with MycP_5_ containing a Twin-Strep-tag at its C terminus. Also, this tag did not interfere with MycP_5_ functioning (see Fig. S1D). As we observed that DSP severely interfered with the pulldown, we used formaldehyde to fix protein-protein interactions. This cross-linking agent did not affect the pulldown efficiency, as similar MycP_5_ levels were detected in the elution samples containing the Strep-tagged MycP_5_, whereas no MycP_5_ was detected in the elution of the wild-type samples by immunoblotting and Coomassie staining (see Fig. 4D; see also Fig. S3). The purification of MycP_5_ resulted in the copurification of EccB_5_ and EccE_5_, albeit at relatively low levels (Fig. 4D). This interaction could be stabilized by formaldehyde treatment, as we detected higher levels of EccB_5_ and EccE_5_ in the cross-linked samples (Fig. 4D). We did not detect the unrelated FtsH membrane component or the abundant GroEL2 cytosolic component in the elution samples (Fig. 4D). We did observe a very-low-intensity signal for EccB_1_ in the elution fraction; however, in contrast to EccB_5_ and EccE_5_, this signal was reduced in the cross-linked elution sample, indicating that this represented nonspecific contamination of the eluate. Our data therefore seem to confirm that MycP_5_ indeed interacts with the ESX-5 core complex components and that this interaction is essential for the stability and functionality of the ESX complexes corresponding to their respective mycosins.

## DISCUSSION

In this study, we showed that the active site mutant of MycP_1_ has a phenotype in *M. marinum* that is similar to that previously observed in *M. tuberculosis* (22). In that previous study, Ohol et al. (22) described a regulatory role of the proteolytic activity of MycP_1_ in *M. tuberculosis*, with increased secretion by the MycP active site mutation. This mechanism appears to be a conserved feature, as we also observed increased secretion of EsxA, EsxB, and EspE in an *M. marinum* strain harboring a proteolytic inactive MycP_1_.

We used the ability of *M. marinum* to lyse erythrocytes in an ESX-1-dependent manner, to further analyze ESX-1 functioning. While the *M. marinum* Δ*mycP_1_* mutant was indeed unable to lyse erythrocytes, the active site mutant showed significantly increased hemolytic activity, corresponding to the increased activity of the ESX-1 system. It is possible that this was due to the increased secretion of EsxA, as this substrate has been indicated to be responsible for the hemolytic activity (11, 12, 28), although the other substrates of ESX-1 are also secreted in larger amounts. The disparity between the increased membrane lysing capability observed in *M. marinum* and the decreased virulence of *M. tuberculosis* in mice (22) may be explained by the immunogenicity of EsxA, which might result in reduced virulence in later stages of infection (29, 30).

MycP_5_ showed a phenotype similar to that of MycP_1_, with the mutation in the predicted active site not affecting the secretion of ESX-5 substrates. However, we did not observe increased secretion in the *mycP_5_* active site mutant, supporting the suggestion that the observed phenotype of the proteolytically inactive MycP_1_ is caused by a specific MycP_1_ substrate, which could be EspB (22). We also did not observe any differences in the (possible) processing of ESX-5 substrates in the *mycP_5_* active site mutant compared to the wild-type *M. marinum* strain. This also means that there are currently no MycP_5_ substrates known. Therefore, the possibility remains that MycP_5_ is proteolytically inactive, although it contains all the features known to be essential for protease activity. We prefer the hypothesis that the phenotype of the active site mutant of *mycP_5_* is a result of functional redundancy between MycP_5_ and other proteases, possibly other mycosins.

We also investigated whether substrate binding is involved in the essential role of mycosins by introducing a bulky amino acid in the substrate binding pocket of MycP_1_ and MycP_5_. Because these modifications had an effect similar to that seen with the active site mutations, we can conclude that not only the proteolytic activity of mycosins but also substrate binding is not required for ESX-dependent secretion. It should be mentioned, though, that the mutated residue of MycP_1_, N239, coordinates the oxyanion hole and, as such, may also affect proteolytic activity. Further experiments are required to determine whether EspB indeed cannot bind to MycP_1mth_::N239Y.

To study the involvement of mycosin in T7S membrane complex functioning, we isolated both the ESX-1 membrane complex and the ESX-5 membrane complex using a Twin-Strep-tag that was fused at the C terminus of EccC. The Strep-tag purification considerably increased the yield and purity of the purified ESX-5 complex compared to the previous purifications using antibodies (2). Despite the improved purification, we were still unable to detect any additional (less-abundant) components; although a few spectral counts of MycP_5_ were specifically detected with the EccC_5_-Strep pulldown, these were not above the spectral count threshold levels. We also were unable to detect specific MycP_5_ copurification by immunoblot analysis using MycP_5_ antibodies. Also, in the Strep-pulldown experiments of the ESX-1 complex we could detect a few specific spectral counts for MycP_1_, but these numbers were again below the threshold level.

We calculated the normalized spectral abundance factor (NSAF) of the Strep-tag-purified complexes using a method similar to a method described before (2) to estimate and compare the relative abundances of individual components of the ESX-1 and ESX-5 complexes. For this, the number of spectral counts (SpC) per isolated protein was divided by the protein’s length (L), which was again divided by the result of SpC/L for all isolated proteins in the experiment. This analysis revealed an EccC_5_/EccB_5_/EccE_5_/EccD_5_ ratio of approximately 2:1:1:1. This ratio is slightly different from the 2:2:1:2 ratio that was found for the antibody pulldown (2). It should be noted that EccC_5_ might be overrepresented in the Strep pulldown results, as this component contains the affinity tag. The NSAF values of the ESX-1 purified proteins revealed a ratio of 9:7:4:4:1 for EccCa_1_/EccCb_1_/EccB_1_/EccE_1_/EccD_1_, showing a similar distribution, in which the EccC subunits, which are produced as two separate proteins in ESX-1, are present at roughly double the amount seen with the other components. For ESX-1, only EccD_1_ seemed to be underrepresented compared to ESX-5. This could suggest that the ESX-1 complex is less stable than the ESX-5 complex, which could also explain the smearing pattern observed in BN-PAGE.

As MycP_1_ or MycP_5_ does not appear to be a (stable) component of the ESX-1 or ESX-5 complex in *M. marinum*, it was surprising that the presence of both MycP_1_ and MycP_5_ is required for complex stability. The instability of the ESX-5 complex in the *mycP_5_* knockout was further indicated by the observation that we could not stabilize the ESX-5 complex by cross-linking in the *mycP_5_* knockout background after repeated freeze-thaw cycles, while this was possible with wild-type samples (unpublished observations). The mechanical stress of this process is apparently already sufficient to dissociate this unstable complex. Using a cross-linking approach, we showed that the ESX-1 complex and the ESX-5 complex could be formed in the *mycP_1_* and *mycP_5_* knockout strains, respectively. Therefore, this indicates that the mycosins are associated with the complexes and are essential for their stability. Furthermore, this stabilization is required for the complex to be functional. The fact that we are unable to detect MycP_1_ or MycP_5_ above the threshold levels in the EccCb_1_ and EccC_5_ pulldown experiments indicates that MycP associates with the membrane complex only loosely and that its interaction is not maintained after detergent extraction. This notion is supported by the observed shift of HA-tagged MycP_5_ to a molecular weight corresponding to the ESX-5 complex on BN-PAGE, the detection of EccB_5_ and EccE_5_ in the elution samples from Strep-Tactin pulldown experiments using Strep-tagged MycP_5_, and the fact that we observe increased amounts of copurified EccB_5_ and EccE_5_ after cross-linking. From this, we conclude that there is indeed an interaction between MycP_5_ and the ESX-5 core complex, which could explain the observed effects on complex stability. Although we cannot explain the exact mechanism by which a loose association of MycP with the core ESX complex can affect the stability of the complex, there are comparable effects known, as reported in the literature. In type IV pilus biogenesis in *Neisseria meningitides*, for instance, the outer membrane protein PilW stabilizes multimeric PilQ, the outer membrane secretin, even though PilW is not part of the multimeric complex formed by PilQ (31).

**FIG 5:**
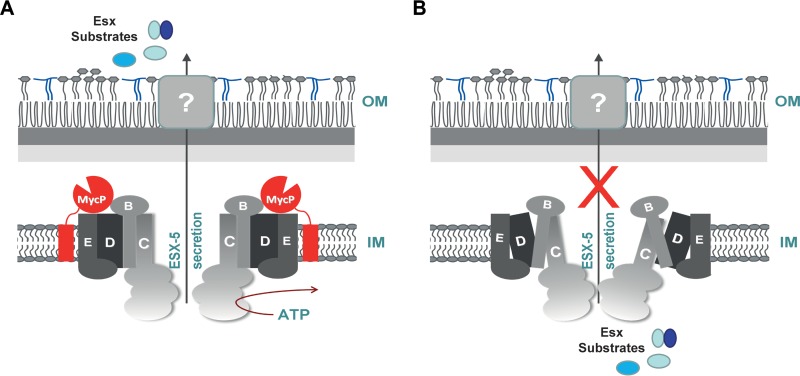
Model of the T7S membrane complex. (A) MycP_5_ associates with the EccBCDE_5_ (B/C/D/E) membrane-embedded complex, and, as a result, the T7S ESX-5 complex is stabilized and functional. IM, inner membrane; OM, outer membrane. (B) In the absence of MycP_5_, the ESX-5 complex is less stable and, as a result, nonfunctional.

In summary, this study for the first time provided insight into the essential function of mycosins in the T7S system. We propose a new model for the T7S systems in mycobacteria, with the mycosins being associated with their respective membrane complexes, which is crucial for the full integrity of the core secretion complex (Fig. 5A). In the absence of mycosin, the complex is less stable and, as a result, nonfunctional (Fig. 5B).

## MATERIALS AND METHODS

### Bacterial strains and culture conditions.

*M. marinum* M^USA^ (32) was used for all *M. marinum* experiments unless stated otherwise. *M. marinum* wild-type strains and the various derived knockout mutants were grown on 7H10 agar supplemented with 10% Middlebrook oleic acid-albumin-dextrose-catalase (OADC) (BD Biosciences) at 30°C or in Middlebrook 7H9 liquid medium supplemented with 10% Middlebrook ADC and, when required, 0.05% Tween 80 at 30°C and 150 rpm. *Escherichia coli* strains were grown in Luria-Bertani (LB) liquid medium or on LB agar. Medium was supplemented with the appropriate antibiotics at the following concentrations: kanamycin, 25 µg ml^−1^; hygromycin, 50 µg ml^−1^; streptomycin, 35 µg ml^−1^; ampicillin, 100 µg ml^−1^; chloramphenicol, 30 µg ml^−1^. *E. coli* strain DH5α was used for DNA cloning and plasmid accumulation and *E. coli* strain Rosetta for recombinant protein expression.

### Generating the *mycP_1_* knockout in *M. marinum.*

The generation of the *mycP_5_* and *eccC_5_* knockout strains that were used in this study was described previously by Ates et al. (4). Notably, a pSMT3 plasmid expressing the outer membrane porin MspA was present in these ESX-5 mutants to circumvent the essentiality of this system for growth of *M. marinum*. The *mycP_5_* knockout mutant did not show a growth defect in the presence of MspA. A *mycP_1_* knockout was created in *M. marinum* M^USA^ by allelic exchange using the phAE159 temperature-sensitive phage (33) and a method similar to that described for the creation of the *mycP_5_* and *eccC_5_* knockout by Ates et al. (4). The required construct was made by DNA amplification using primers MycP1 LF, MycP1 LR, MycP1 RF, and MycP1 RR (see Table S1 in the supplemental material) and the in-Fusion enzyme. The chromosomal deletion was confirmed by PCR analysis and sequencing. The *M. marinum* E11 *eccCb_1_* transposon mutant that was used in this study has been described previously by Stoop et al. (27).

### Cloning.

The *mycP_1_* and *mycP_5_* genes were amplified from *M. marinum* M^USA^ genomic DNA by PCR with anchored primers (EcoRI and HindIII; see Table S1 in the supplemental material). Point mutations, the HA tag, and Twin-Strep-tag were introduced into *mycP_1_* and *mycP_5_* with nested primers (see Table S1). The generated constructs were additionally cloned as EcoRI-HindIII-digested fragments in pMV361 (34) or with PmII and HindIII in the case of the Twin-Strep-tag. The C-terminal Twin-Strep-tag was introduced into EccC_5_ by modifying the pMV-EccBC_5_ vector described by Ates et al. (4). The vector was digested with DraI and HindIII, and a linker consisting of two annealed oligonucleotides (see Table S1 [OneStrep-1 and OneStrep-2]) was subsequently ligated to the digested vector. For the generation of pMV-EccBCab_1_-Twin-Strep, the *eccBCab_1_* genes were amplified from *M. marinum* E11 genomic DNA with two consecutive PCRs. The first PCR amplified *eccBCab_1_* with an additional 100 to 200 bp on both sides of each gene, and the second PCR amplified *eccBCab_1_* and introduced a NsiI site in front of the gene (see Table S1). The PCR product was digested with NsiI and ligated into DraI- and NsiI-digested pMV-EccBC_5_-Twin-Strep. MycP_1mth_, MycP_1mth_ S334A, and EspB_mtb_ *E. coli* expression plasmids were previous described by Wagner et al. (25). The *mycP_1mth_*::*N239Y* construct was amplified with anchored primers (NdeI and XhoI) using pET-21d-*mycP_1mth_* as a template, and the point mutation was introduced with nested primers (see Table S1). The construct was digested with NdeI and XhoI and ligated to NdeI- and XhoI-digested pet-28a. All plasmids were checked by sequencing of the relevant sections.

### Protein secretion and immunoblot analysis.

*M. marinum* strains were grown in 7H9 liquid medium supplemented with ADC, 0.05% Tween 80, and appropriate antibiotics until mid-logarithmic phase, after which the cells were washed and inoculated in 7H9 medium with 0.2% dextrose–0.05% Tween 80 at an optical density at 600 nm (OD_600_) of 0.4 and grown for another 16 h. The cells (Pellet) were spun down for 10 min at 6,000 × *g*, washed with phosphate-buffered saline (PBS), and resuspended in SDS loading buffer. Supernatants were passed through 0.45 -µm-pore-size filter units, and proteins were precipitated with trichloroacetic acid (TCA) and resuspended in SDS loading buffer. Alternatively, the cells were resuspended in 0.5% Genapol X-080 and incubated for 1 h at room temperature. Samples were spun down and pellets were resuspended in SDS sample loading buffer (Genapol Pellet), while 5× SDS sample buffer was added to the supernatant containing Genapol X-080 (Genapol Supernatant). Proteins were separated on SDS-PAGE gels and transferred to a nitrocellulose membrane, and membranes were stained with anti-GroEL2 (Cs44; John Belisle, NIH, Bethesda, MD, USA), anti-PE_PGRS (31), anti-ESAT-6 (monoclonal antibody [MAb] Hyb76-8), anti-HA (HA.11; Covance), anti-EccB5 (2), anti-EccE5 (2), anti-EspE (Eric Brown; Genentech), anti-EsxN (Mtb9.9) (35), anti-CFP-10 (Colorado State University), anti-EspB (EPFL, Lausanne, Switzerland), or anti-FtsH (36) antibodies. Polyclonal antiserum against the EccB_1_ synthetic peptide CLPSDPNPRKVPAG was raised in rabbits by Innovagen (Lund, Sweden) using Stimune (Prionix) as an adjuvant.

### Protein expression and purification and activity assays.

Recombinant proteins were expressed in *E. coli* Rosetta (DE3) cells by induction with 0.5 mM IPTG (isopropyl-β-d-thiogalactopyranoside) for 4 h at 22°C. Cells were harvested and resuspended in 20 mM Tris-HCl (pH 8.0)–300 mM NaCl. The cells were lysed using lysozyme (1 µg ml^−1^) and a One Shot cell disruptor (Constant Systems Ltd.). The cell lysate was centrifuged for 20 min at 8,000 × *g*, and the proteins were purified from the cleared supernatant with a HiTrap Talon crude column (GE Life Sciences), using an elution gradient of 0 mM to 250 mM imidazole. Purified proteins were dialyzed using 20 mM HEPES (pH 7.5)–100 mM NaCl. Mycosin activity assays were performed using 20 mM HEPES (pH 7.5)–100 mM NaCl–2 mM CaCl_2_–5 mM FeCl–5 mM MgCl at 37°C for 16 h with 0.2 mg ml^−1^ EspB and 0.1 mg ml^−1^ mycosin. Reactions were stopped by the addition of SDS loading buffer. Samples were heated at 94°C for 5 min, and the proteins were separated on a 10% SDS-PAGE gel. Proteins were visualized by Coomassie staining or by immunoblotting, using mouse anti-His antibodies (GE Healthcare).

### Hemolysis.

Mid-log-phase *M. marinum* bacteria were harvested by centrifugation, washed with PBS, and resuspended in phenol red-free Dulbecco’s modified Eagle’s medium (DMEM) (Gibco). Bacteria from all strains were set to a concentration of 2 OD units ml^−1^. Defibrinated sheep blood cells (Oxoid) were washed with DMEM and set to a concentration of 8 × 10^8^ cells ml^−1^. A 75-μl volume of bacteria and 75 µl of erythrocytes were mixed and spun down for 5 min at 610 × *g* in a round-bottom, 96-well plate. The bacteria and cells were incubated in a 5% carbon dioxide incubator at 32°C for 3 h. The pellets were resuspended and repelleted, the supernatant was transferred to a flat-bottom, 96-well plate, and the released hemoglobin was quantified by the measured absorbance at 405 nm. Statistically significant differences between strains were determined with one-way analysis of variance (ANOVA). The sample size consisted of 6 biological replicates per strain, with each biological replicate consisting of 4 technical replicates.

### Blue native PAGE analysis of ESX membrane complex formation.

*M. marinum* bacteria were grown to an OD_600_ of 1 to 1.5 and harvested by centrifugation. Cells were resuspended in PBS–250 mM sucrose and lysed with a One Shot cell disruptor (Constant Systems Ltd.). Unlysed cells were pelleted by centrifugation at 3,000 × *g* for 10 min. The cell envelope (CE) fraction was isolated by centrifugation at 100,000 × *g* for 30 min and resuspended in PBS–250 mM sucrose. Where stated, samples were cross-linked with DSP or were treated with dimethyl sulfoxide (DMSO) as a negative control and were subsequently quenched with 100 mM glycine–10 mm NaHPO_4_ (pH 8.5). Membrane proteins were solubilized for 1 h with 0.25% DDM, the insoluble fraction was removed by centrifugation at 100,000 × *g* for 20 min, and solubilized proteins (in complexes) were separated on a 3% to 12% NativePage Novex bis-Tris protein gel (Life Technologies). Proteins were transferred to a polyvinylidene difluoride (PVDF) membrane and stained with anti-EccB_1_, anti-EccB_5_, anti-EccC_5_ (2), anti-EccD_5_ (2), or anti-HA antibodies.

### Isolation of ESX-1 and ESX-5 membrane complexes and MycP_5_ pulldown.

Proteins were solubilized from isolated CE fractions as described above, with the addition of 0.3 mg/ml avidin (Sigma) after the DDM incubation. Solubilized proteins were incubated with Strep-Tactin beads for 30 min in a head-over-head manner, washed with 50 mM HEPES-KOH (pH 7.8)–150 mM KOAc–125 mM sucrose–0.04% DDM, and eluted with 10 mM desthiobiotin, dissolved in the same buffer as was used for the washing. For the MycP_5_-Strep-tag purification, where stated, whole cells were treated with 1% formaldehyde and subsequently quenched with 100 mM glycine–10 mm NaHPO_4_ (pH 8.5). Proteins were solubilized from isolated CE fractions as described above, and the Strep-Tactin pulldown was performed as described above. SDS solubilization buffer was added to the elution fractions, and samples were heated at 94°C, separated on a 10% SDS-PAGE gel, and visualized by Coomassie staining or transferred to a nitrocellulose membrane and stained with anti-Strep-tag, anti-EccB1, anti-EccB_5_, anti-EccD_5_, or anti-EccE_5_ (2) antibodies. SDS solubilization buffer was added to the elution fractions, and samples were heated at 94°C, separated on a 12.5% SDS-PAGE gel, and visualized by Coomassie staining or transferred to a nitrocellulose membrane and stained with anti-Strep-tag, anti-EccB_1_, anti-EccB_5_, anti-FtsH, anti-GroEL2, or anti-EccE_5_ (2) antibodies.

### LC-MS/MS.

Peptides were separated by the use of an UltiMate 3000 nanoLC-MS/MS system (Dionex LC-Packings, Amsterdam, the Netherlands) equipped with a 20-cm-by-75-µm-inner-diameter (ID) fused-silica column custom packed with 3-µm-diameter 120-Å reprosil Pur C_18_ aqua (Dr. Maisch GmbH, Ammerbuch-Entringen, Germany). After injection, peptides were trapped at 6 µl/min on a 10-mm-by-100-µm-ID trap column packed with 5 µm 120-Å reprosil Pur C_18_ aqua using 2% buffer B (buffer A, 0.5% acetic acid–Milli-Q Water [MQ]; buffer B, 80% Acetonitrile [ACN]–0.5% acetic acid–MQ) and separated at 300 nl/min in a 10% to 40% buffer B gradient in 60 min (90 min, injection to injection). Eluting peptides were ionized at a potential of +2 kVA into a Q Exactive mass spectrometer (Thermo, Fisher, Bremen, Germany). Intact masses were measured at a resolution of 70,000 (at *m*/*z* 200) in the Orbitrap using an automatic gain control (AGC) target value of 3 × 10^6^ charges. The top 10 peptide signals (charge states 2^+^ and higher) were submitted to MS/MS in the high-cell-density (HCD) (higher-energy collision) cell (4-amu isolation width, 25% normalized collision energy). MS/MS spectra were acquired at a resolution of 17,500 (at *m*/*z* 200) in the Orbitrap using an AGC target value of 2 × 10^5^ charges and an underfill ratio of 0.1%. Dynamic exclusion was applied with a repeat count of 1 and an exclusion time of 30 s.

MS/MS spectra were searched against the Uniprot *M. marinum* complete proteome (ATCC BAA-535M) FASTA file (5,418 entries) using MaxQuant 1.4.1.2 (37). Enzyme specificity was set to trypsin, and up to two missed cleavages were allowed. Cysteine carboxamidomethylation (Cys; +57.021464 Da) was treated as a fixed modification and methionine oxidation (Met, +15.994915 Da) and N-terminal acetylation (N terminal, +42.010565 Da) as variable modifications. Peptide precursor ions were searched with a maximum mass deviation of 6.0 ppm and fragment ions with a maximum mass deviation of 20 ppm (default MaxQuant settings). Peptide and protein identifications were filtered at a false-discovery rate (FDR) of 1% using the decoy database strategy. Proteins that could not be differentiated based on MS/MS spectra alone were grouped into protein groups (default MaxQuant settings).

Proteins were quantified (in a label-free manner) by spectral counting, i.e., by determining the sum of all MS/MS spectra for each identified protein (38). For quantitative analysis across samples, spectral counts for identified proteins in a sample were normalized to the sum of spectral counts for that sample. This gives the spectral count contribution of a protein relative to the contribution of all spectral counts in the sample. For comparisons of different biological samples, these normalized spectral counts were used to calculate ratios. In this way, we were able to correct for loading differences between samples. Differential analysis of samples was performed using the beta-binominal test (39), which takes into account within- and between-sample variations, giving fold change values and associated *P* values for all identified proteins. Protein cluster analysis of the differentially expressed proteins was performed using hierarchical clustering in R. The protein abundances were normalized to zero mean and unit variance for each individual protein. Subsequently, the Euclidean distance measure was used for protein clustering.

### Accession number(s).

The mass spectrometry proteomics data have been deposited in the ProteomeXchange Consortium via the PRIDE (40) partner repository with the data set identifier PXD003766.

## SUPPLEMENTAL MATERIAL

Figure S1  Introduction of a Twin-Strep-tag or HA tag at the C terminus of EccC_b1_, EccC_5_, or MycP_5_ does not interfere with ESX-dependent secretion. (A) Immunoblot analysis of supernatants and cell pellets of the *M. marinum* wild-type (WT) strain, an *eccCb_1_* transposon mutant (tn::*eccCb1*), and the Strep-tagged complemented *eccCb_1_*::*tn-eccCb_1_-2strep* (EccCb1-Strep) mutant. GroEL2 staining was used as a control for lysis and equal loading. (B) Immunoblot analysis of supernatants and cell pellets of the *M. marinum* wild-type (WT) strain, an *eccC_5_* deletion mutant (Δ*eccC_5_*), and the Strep-tagged complemented *ΔeccC_5_*-*eccC_5_-2strep* (EccC5-Strep) mutant. GroEL2 staining was used as a control for lysis and equal loading. (C) Immunoblot analysis of cellular proteins (genapol pellet, GP) and cell surface-localized (genapol supernatant, GS) proteins of the *M. marinum* wild-type (WT) strain and the HA-tagged complemented *ΔmycP_5_-mycP_5_-HA* (*MycP5-HA*) mutant. GroEL2 staining was used as a control for lysis and equal loading. (D) Immunoblot analysis of cellular (GP) and cell surface-localized (GS) proteins of *the M. marinum* wild-type (WT) strain and the Strep-tagged complemented Δ*mycP_5_-mycP_5_-2Strep* (*MycP5-Strep*) mutant. GroEL2 staining was used as a control for lysis and equal loading. Download Figure S1, TIF file, 1.9 MB

Figure S2  MycP_1_ and MycP_5_ are essential for ESX membrane complex stability. (A) Immunoblot analysis of detergent-solubilized cell envelope fractions of wild-type (WT) *M. marinum* and the *mycP_5_* deletion strain complemented with various *mycP_5_* mutant genes after BN-PAGE (BN) or SDS-PAGE (SDS). Blots were incubated with antibodies directed against EccC_5_ and EccD_5_. (B) Immunoblot analysis of DSP-cross-linked (+DSP) or DMSO-only-treated (−DSP) detergent-solubilized cell envelope fractions of the *M. marinum* wild-type (WT) strain, an *eccCb1*::*tn* mutant, and a *mycP1* deletion mutant, stained for EccB_1_ after BN-PAGE (BN) or for FtsH after SDS-PAGE (SDS). Download Figure S2, TIF file, 0.8 MB

Figure S3  Purified Strep-tagged MycP_5_ is at observable levels after Coomassie brilliant blue (CBB) staining. Results of SDS-PAGE analysis and Coomassie staining in Strep-Tactin pulldown experiments using the *M. marinum* WT (WT) strain and the *ΔmycP_5_*::*mycP_5_-Strep* (MycP5-Strep) mutant are shown. The same background bands are visible in the MycP_5_-Strep and WT samples. Download Figure S3, TIF file, 0.2 MB

Table S1  List of primers used in this study. Table S1, DOCX file, 0.01 MB
