# Supplementary figures and images for: Mycosins Are Required for the Stabilization of the ESX-1 and ESX-5 Type VII Secretion Membrane Complexes
Source: mBio. 2016 Oct 18;7(5):e01471-16. doi: 10.1128/mBio.01471-16 (PMC5082899; doi:10.1128/mBio.01471-16)

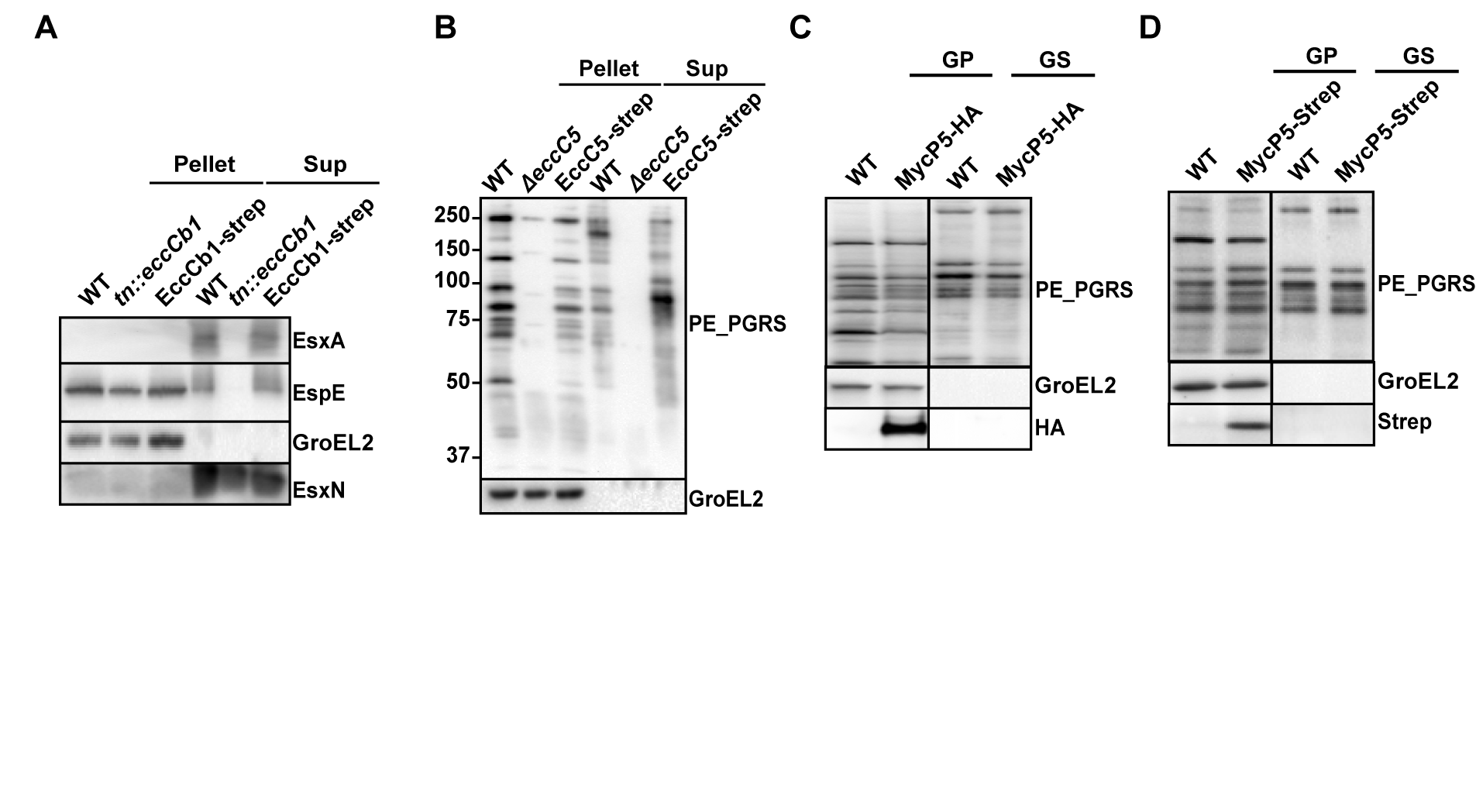

Supplement: Figure S1 — Introduction of a Twin-Strep-tag or HA tag at the C terminus of EccCb1, EccC5, or MycP5 does not interfere with ESX-dependent secretion. (A) Immunoblot analysis of supernatants and cell pellets of the M. marinum wild-type (WT) strain, an eccCb1 transposon mutant (tn::eccCb1), and the Strep-tagged complemented eccCb1::tn-eccCb1-2strep (EccCb1-Strep) mutant. GroEL2 staining was used as a control for lysis and equal loading. (B) Immunoblot analysis of supernatants and cell pellets of the M. marinum wild-type (WT) strain, an eccC5 deletion mutant (ΔeccC5), and the Strep-tagged complemented ΔeccC5-eccC5-2strep (EccC5-Strep) mutant. GroEL2 staining was used as a control for lysis and equal loading. (C) Immunoblot analysis of cellular proteins (genapol pellet, GP) and cell surface-localized (genapol supernatant, GS) proteins of the M. marinum wild-type (WT) strain and the HA-tagged complemented ΔmycP5-mycP5-HA (MycP5-HA) mutant. GroEL2 staining was used as a control for lysis and equal loading. (D) Immunoblot analysis of cellular (GP) and cell surface-localized (GS) proteins of the M. marinum wild-type (WT) strain and the Strep-tagged complemented ΔmycP5-mycP5-2Strep (MycP5-Strep) mutant. GroEL2 staining was used as a control for lysis and equal loading. Download [file mbo005163033sf1.tif]

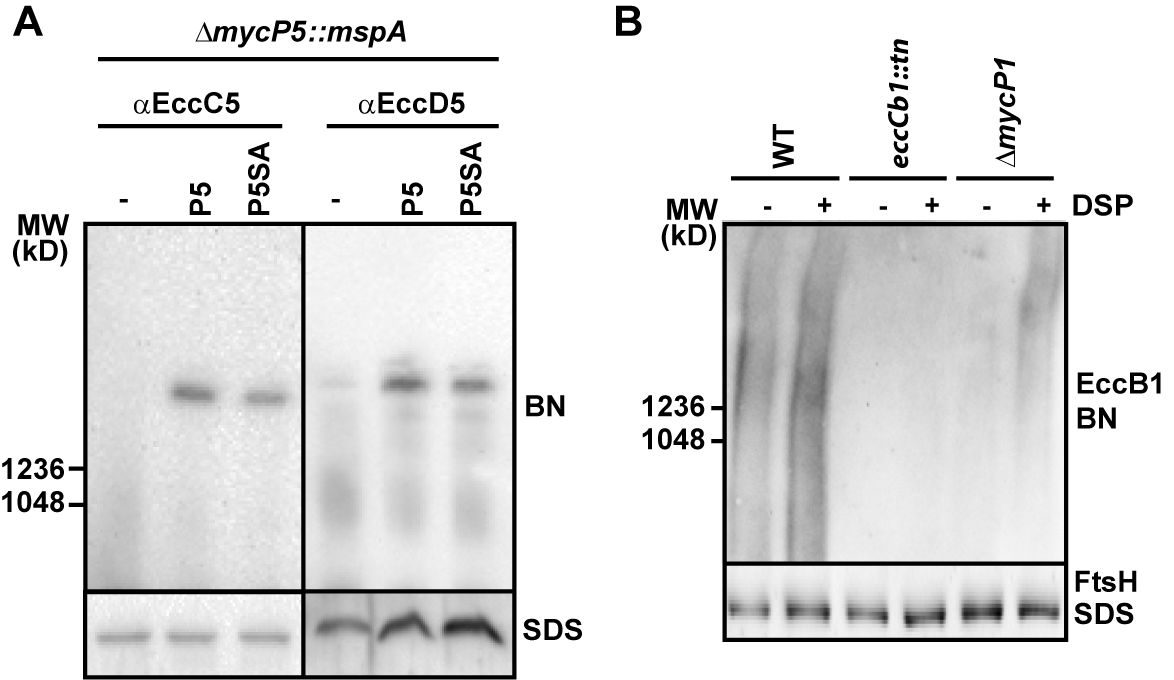

Supplement: Figure S2 — MycP1 and MycP5 are essential for ESX membrane complex stability. (A) Immunoblot analysis of detergent-solubilized cell envelope fractions of wild-type (WT) M. marinum and the mycP5 deletion strain complemented with various mycP5 mutant genes after BN-PAGE (BN) or SDS-PAGE (SDS). Blots were incubated with antibodies directed against EccC5 and EccD5. (B) Immunoblot analysis of DSP-cross-linked (+DSP) or DMSO-only-treated (−DSP) detergent-solubilized cell envelope fractions of the M. marinum wild-type (WT) strain, an eccCb1::tn mutant, and a mycP1 deletion mutant, stained for EccB1 after BN-PAGE (BN) or for FtsH after SDS-PAGE (SDS). Download [file mbo005163033sf2.tif]

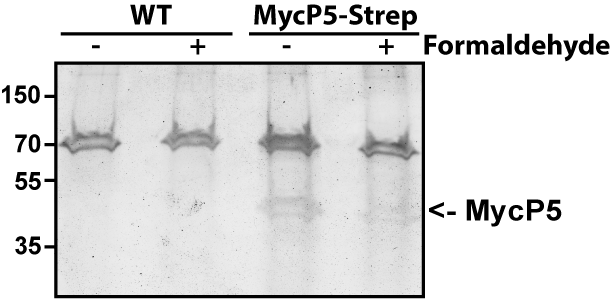

Supplement: Figure S3 — Purified Strep-tagged MycP5 is at observable levels after Coomassie brilliant blue (CBB) staining. Results of SDS-PAGE analysis and Coomassie staining in Strep-Tactin pulldown experiments using the M. marinum WT (WT) strain and the ΔmycP5::mycP5-Strep (MycP5-Strep) mutant are shown. The same background bands are visible in the MycP5-Strep and WT samples. Download [file mbo005163033sf3.tif]
