# Supplementary material for: Mycosins Are Required for the Stabilization of the ESX-1 and ESX-5 Type VII Secretion Membrane Complexes
Source: mBio. 2016 Oct 18;7(5):e01471-16. doi: 10.1128/mBio.01471-16 (PMC5082899; doi:10.1128/mBio.01471-16)
Supplement: Table S1 — List of primers used in this study. [file mbo005163033st1.docx]

**Table S1.** List of primers used in this study

| **Name** | **Sequence (5’ 🡪 3’)** |
| --- | --- |
| MYCP1 LF | tgtcaaacctgccaaccgattggcagttggcagtg |
| MYCP1 LR | caggactctagccaaccggaggcggtatcgacaat |
| MYCP1 RF | tacaggacctgccaattaaatagccgcagtgaagctctgg |
| MYCP1 RR | tacgactcactccaaggcatgcgcgttcatcaact |
| MYCP1ECORI FW | ccggaattccatatgcaggcaggactgacac |
| MYCP1HINDIII REV | gccgaagctttcatcggcgcctcagcg |
| MYCP5ECORI FW | ccggaattccat atgcagcgattcggtaccgtt |
| MYCP5HINDIII REV | gccgaagctt tcatcgccgcttccgtga |
| ECCBC1 FW NEST | ccgatatgcgcgacaattgg |
| ECCBC1 REV NEST | gagcgttgccgctcaatagt |
| ECCBC1 NSII FW | ttttatgcataggcaatcgccacggtgcat |
| ECCBC1 REV (BLUNT) | accggggcttgggggtgctgc |
| ONESTREP-1 | aaaagcgcttggagccacccgcagttcgagaagggaggaggttcgggaggtggatcgggaggtggatcgtggagccacccgcagttcgagaagtaa |
| ONESTREP-2 | agctttacttctcgaactgcgggtggctccacgatccacctcccgatccacctcccgaacctcctcccttctcgaactgcgggtggctccaagcgctttt |
| MYCP1S354A FW | cccgatcgccggcaccgcatttgcggcggcctatgtc |
| MYCP1S354A REV | gacataggccgccgcaaatgcggtgccggcgatcggg |
| MYCP1N259Y FW | ggtcgtcgccgcgggttacaccggcggcgactgctc |
| MYCP1N259Y REV | gagcagtcgccgccggtgtaacccgcggcgacgacc |
| MYCP5-STREP FW | ggcgatcacggaagcggcgaagcgcttggagccacccgca |
| MYCP5-STREP REV | tgcgggtggctccaagcgcttcgccgcttccgtgatcgcc |
| MYCP5D362Y FW | cggccggctacggcagcaaga |
| MYCP5D362Y REV | tcttgctgccgtagccggccg |
| ESPBNHEI FW | ccggctagcatgagccagccgcagaccgt |
| ESPBHABAMHI REV | ggcggatccactacgcgtagtccggcacgtcgtacgggtacttgttgtcctgacggcg |
| MYCP1MTHNDEI FW | ccgcatatgatcgaacctccggtga |
| MYCP1MTHXHOI REV | gccgctcgagctacacatcccaggtcagcgc |
| MYCP1MTHN239Y FW | gatcgcggcggccggctacaccggtcaggactgcacc |
| MYCP1MTHN239Y REV | ggtgcagtcctgaccggtgtagccggccgccgcgatc |
| MYCP5HA REV | gccgaagcttctacgcgtagtccggcacgtcgtacgggtatcgccgcttccgtga |
